# Design of a cosmetic glove stiffness compensation mechanism for toddler-sized hand prostheses

**DOI:** 10.1371/journal.pone.0183233

**Published:** 2017-08-11

**Authors:** Ronald A. Bos, Dick H. Plettenburg

**Affiliations:** Department of Biomechanical Engineering, Delft University of Technology, Delft, The Netherlands; Northwestern University, UNITED STATES

## Abstract

The addition of a cosmetic glove to an upper limb prosthesis has a distinct effect on the cosmetic value, but its viscoelastic behaviour adds a substantial amount of stiffness and hysteresis to the system. As a result, the overall usability of the prosthesis is degraded. A novel negative stiffness element is designed to compensate for the cosmetic glove's stiffness. A combination of linear helical springs and the concept of rolling link mechanisms has resulted in a Rolling Stiffness Compensation Mechanism (RSCM). Results show that the RSCM is capable of exerting a progressive negative stiffness characteristic and can be built small enough to fit inside a 33 mm diameter wrist. Using the RSCM, an otherwise voluntary opening toddler-sized prosthesis is converted into a voluntary closing device, reducing maximum operation forces down to 40 N with a combined efficiency of 52%. Further adjustments to the design are possible to further improve the efficiency of the mechanism. Moreover, changes in geometric relations of the mechanism offers possibilities for a wide range of prostheses and other applications.

## Introduction

Missing [parts of] an upper limb has dramatic consequences on a person’s physical abilities and mental health [1–3]. Use of a prosthesis aims to improve those factors and can do so with a perfect fulfilment of the three main requirements in prosthesis design: cosmesis, comfort and control [4]. However, reports show that many users are dissatisfied with their prosthesis and rejection rates range from 20%–40% [5]. Overuse injuries to the remaining limb, as well as back- and neck-pain often account for rejection. If not rejected, active prostheses sometimes end up being used only for cosmetic reasons [4–6]. Clearly, current prostheses do not meet all requirements.

Among body-powered hand prostheses the mechanical efficiency is low, whereas hooks often show much better performances [7–10] The activation forces for hands are high and range from 60–130 N for a small 15 N pinch force [10], while the comfortable limit is at 40 N [11]. High activation forces also disturb the proprioceptive feedback [11]. The addition of a cosmetic glove is the most prominent cause of these high forces. The viscoelastic behaviour of the material adds a large amount of stiffness and energy losses due to hysteresis [12–15]. Moreover, it limits the usability of voluntary closing devices (a prosthesis that closes upon activation by the user), which are advantageous due to their increased mechanical efficiency and proprioceptive feedback compared to their voluntary opening counterparts [8, 16]. This problem increases in severity for toddler-sized prostheses, as the relative thickness of the glove increases and children are less capable of producing high activation forces [17]. It appears that the desire for a natural looking hand, i.e. improved cosmesis, counteracts the comfort and control of the prosthesis–being the main advantages of a body-powered prosthesis.

Several solutions are possible in order to address this problem:

- cosmetic glove omission;
- cosmetic glove modification;
- prosthesis modification; or,
- cosmetic glove stiffness compensation.

Because the cosmetic glove is indispensable[18], alternative materials are very hard to find [19–23], and glove and prosthesis modification can only provide for partial solutions [12], compensation of the glove stiffness is left as solution. This ideology has already lead to a series of mechanisms and methods [12–14, 24–26], but none of them have resulted in a working concept due to challenges that lie in the non-linear behaviour of the glove stiffness, the high occurring forces, and the small working volumes.

This study aims to design a new glove stiffness compensation mechanism for a toddler-sized hand prosthesis. The goal is to reduce activation forces to a minimum and to fit the mechanism into the wrist of the prosthesis. Consequently, emphasis is put on reducing energy dissipation within the mechanism and maximising energy density.

## Methods and materials

### Design criteria

The main objective for the compensation mechanism was to reduce activation forces down to the comfortable limit of 40 N [11]. Furthermore, it should passively open the prosthesis and create a voluntary closing device, due to the advantages in enhanced feedback. A toddler-sized WILMER WHD-4 prosthesis, with a mass of 69 g, was used as a reference point [27]. Consequently, the compensation mechanism needed to be light-weight and fit into a compatible wrist, which may range from 30–38 mm in diameter [28]. At such small scale, the use of hinged and sliding joints becomes impractical and can introduce coefficients of friction of up to *f* = 0.02. For this reason, the concept of rolling link mechanisms was used, which is a method that uses only rolling friction and is able to reduce the friction coefficient down to *f* < 0.001 [24]. The prosthesis operates by using a central pushrod, which allowed for a voluntary opening or voluntary closing device by adding or omitting an extra lever, respectively.

### Design approach

**Fig 1:**
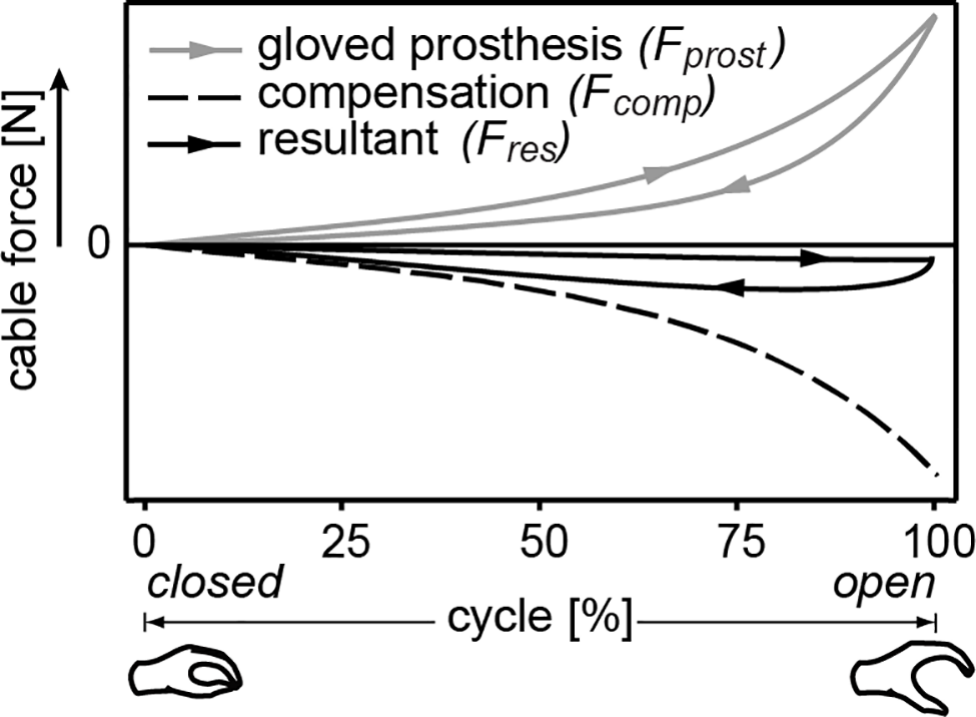
An ideal case of overcompensation of the glove characteristic. The operation of the resultant characteristic is reversed and turns an otherwise voluntary opening device into a voluntary closing device.

The increased stiffness of the cosmetic glove combined with the prosthesis can be compensated by adding a negative stiffness element in parallel to the prosthesis mechanism, such that the addition of the two gives a reduced, resultant stiffness. A prosthesis with cosmetic glove generally possesses a progressive stiffness characteristic [12–14]. By mimicking the shape of this stiffness characteristic, but acting in opposite direction, the resultant stiffness can be reduced to a minimum. Moreover, a voluntary closing device can be acquired with overcompensation (Fig 1).

Merely using a pre-tensioned spring as a negative stiffness element does not suffice, because the compensation force would decrease as the spring releases energy. The opposite is necessary: the compensation force should increase as energy is released from the compensation mechanism. As a result, the design of the mechanism was dependent on three key components: the desired stiffness characteristic, the method of energy storage and the used mechanical linkage.

#### Desired stiffness characteristic

The desired stiffness characteristic of the compensation mechanism was dependent on the used combination of prosthesis and cosmetic glove. Consequently, a total of ten equally sized paediatric gloves were fitted on the WILMER WHD-4 prosthesis and the resulting stiffness characteristic was measured. The combination that resulted in the lowest energy requirement was used for further dimensioning of the compensation mechanism. Six gloves were made of PVC (Otto Bock, size 8S6 = 142x50, thickness of 0.8–1.3 mm), two were made of silicone (Otto Bock, size 8S6 = 142x50, thickness of 2.2–2.5 mm) and two were made of silicone with applied smooth-coating (Regal, Model SC-101L-M-L-CS-2, thickness of 2.8–3.0 mm). Unfortunately, the Otto Bock silicone gloves are not available anymore. All gloves caused the prosthesis to close in rest.

#### Energy storage

Because weight and volume needed to be minimised, energy density was the foremost factor in determining the ideal form of energy storage. Also, the working principle needed to be passive to conserve the body-powered ideology. Among applicable sources of potential energy, i.e. mechanical springs, gas springs and magnetic forces, linear helical springs were the best option with high energy density (1.21 MJ/m^3^ [29]) and were most practical due to many variations in off-the-shelf products.

#### Mechanical linkage

The main purpose of the mechanical linkage was to create the desired compensation characteristic. This is possible with an asymptotic mechanical enhancement curve, which suppresses force when springs are fully tensed and enhances force when they are almost fully relaxed. This asymptote results in high reaction forces from the energy storage, causing energy dissipation due to friction. In order to minimise these losses, the concept of rolling link mechanisms was used. This increases the mechanism's efficiency and reduces the chance for it to fail. It does, however, introduce difficulties in stabilization and alignment of the different parts.

### Conceptual design

**Fig 2:**
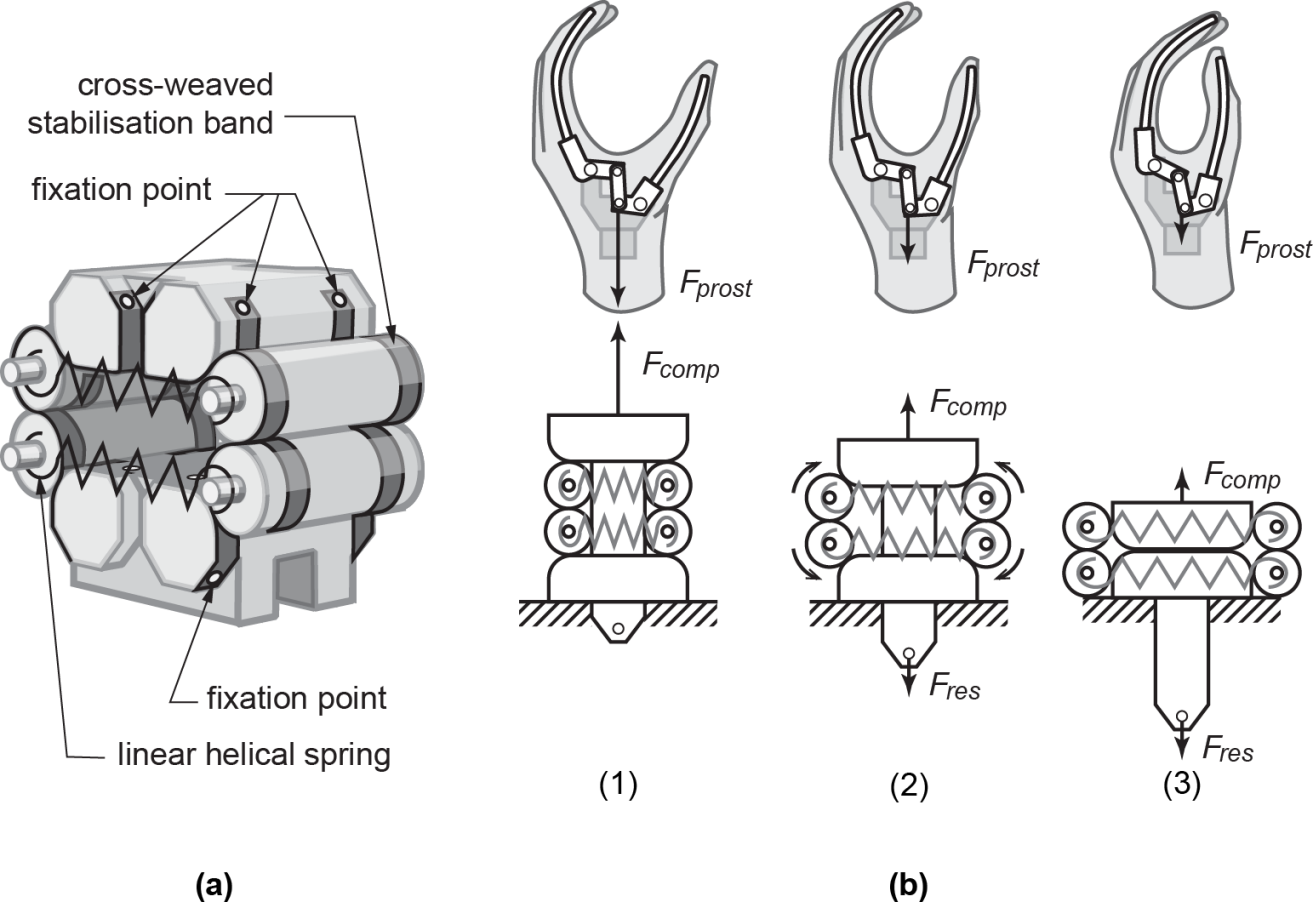
Conceptual design of the Rolling Stiffness Compensation Mechanism (RSCM). (a) Its overall shape and how the parts connect through cross-weaved stabilization bands, indicating the visible fixation points, and (b) its working principle in combination with the WILMER WHD-4 mechanism.

The combination of using linear helical springs as energy storage and a rolling link mechanism as mechanical linkage, led to the design of a Rolling Stiffness Compensation Mechanism (RSCM). The RSCM's overall shape and how the parts connect through stabilization bands is shown in Fig 2A. Its working principle is shown in Fig 2B in three steps, where the force symbols correspond to those in Fig 1. The three steps are:

1. The glove stiffness has the tendency to close the hand, resulting in a force from the gloved prosthesis (*F*_*prost*_). The compensation force from the RSCM (*F*_*comp*_) counteracts this force. Because *F*_*comp*_ *> F*_*prost*_, the hand passively opens.
2. As the user pulls on the RSCM (*F*_*res*_), *F*_*comp*_ decreases and the glove stiffness will cause the hand to close (*F*_*res*_ *+ F*_*prost*_ *> F*_*comp*_). As the hand closes, elastic energy from the cosmetic glove is transferred to the springs in the RSCM.
3. The hand is closed and the glove is relaxed (*F*_*prost*_ *→ 0*). The springs in the RSCM are fully loaded while *F*_*comp*_ is minimal. Increase in *F*_*res*_ will now only increase grip strength. Because *F*_*comp*_ *> F*_*prost*_, the hand will passively open again if *F*_*res*_ becomes zero, returning the system to step (1).

In order for the mechanism to be able to cycle through all these steps in its full range of displacement, two boundary conditions need to be added. Firstly, a mechanical stop is necessary to prevent the rolling elements from rolling inwards during step (1), which would put the mechanism into a form lock. Secondly, strength of grip can only be increased if the intermediate bodies do not touch during step (3).

#### Compensation characteristic

**Fig 3:**
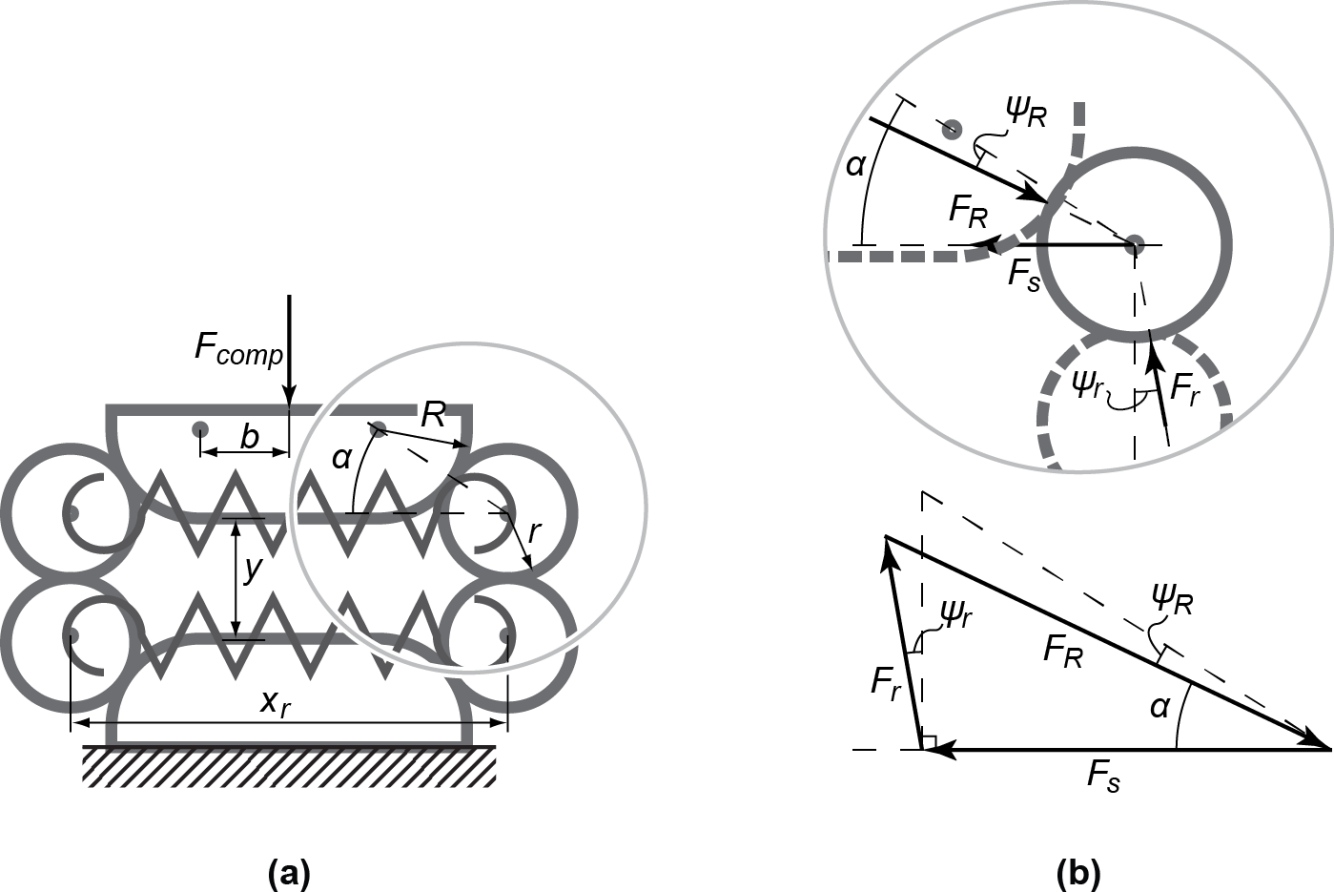
Geometry and force analysis of the mechanism. (a) The used rolling link mechanism and used geometric annotations. In (b), the free body diagram is shown of the roller inside the encircled area, together with the accompanying force triangle in which the forces are in balance. The vertical component of force *F*_*R*_ directly contributes to the total compensation force *F*_*comp*_.

The rolling link mechanism is shown in Fig 3A, which consists of four rollers with radius *r* that are pulled towards each other by linear tensile springs, and two opposing intermediate bodies whose widths are determined by parameter *b* and a contour radius *R*. Due to the horizontal and vertical symmetry of the mechanism, each roller makes the same contact angle *α* with the intermediate bodies. The distance between the rollers' centres is denoted by *x*_*r*_ and determines the spring length, while the vertical distance between the intermediate bodies is denoted by *y* and is directly related to the cable displacement from the prosthesis. They can be calculated as a function of *α* according to:

$$x_{r}(\alpha )=2((R+r)\cos\alpha +b)$$ (1.A)

y(α)=2((R+r)sin⁡α−R+r) (1.B)

In Fig 3B, a free body diagram of one of the rollers is shown. It can be seen that the force from the springs on each roller (*F*_*s*_) induces a reaction force from the adjacent roller (*F*_*r*_) and intermediate body (*F*_*R*_). The magnitude of these reaction forces are dependent on *α*, but the occurrence of free rolling resistance introduces friction angles (*ψ*_*r*_, *ψ*_*R*_) and change the direction and magnitude. In order for all forces to be in balance, the relations as shown in the force triangle need to hold, which can be expressed in the sine rule:

$$\frac{F_{r}}{\sin\left(\alpha -\psi_{R}\right)}=\frac{F_{R}}{\sin\left(\pi /2+\psi_{r}\right)}=\frac{F_{s}}{\sin\left(\pi -(\alpha -\psi_{R})-(\pi /2+\psi_{r})\right)}$$ (2)

Resultantly, *F*_*r*_ and *F*_*R*_ can be calculated:

$$F_{r}(\alpha )=\frac{\sin\left(\alpha -\psi_{R}\right)}{\cos\left(\alpha -\psi_{R}+\psi_{r}\right)}F_{s}$$ (3.A)

$$F_{R}(\alpha )=\frac{\cos\left(\psi_{r}\right)}{\cos\left(\alpha -\psi_{R}+\psi_{r}\right)}F_{s}$$ (3.B)

Additionally, *F*_*s*_ can be expressed in *x*_*r*_ (Eq (1.A)) and the spring's stiffness (*k*), initial length (*L*_*0*_) and pretension (*F*_*0*_). Each roller is connected with two springs, it can therefore be calculated according to:

$$F_{s}(\alpha )=2(k(x_{r}(\alpha )-L_{0})+F_{0})$$ (4)

The compensation force *F*_*comp*_ is dependent on the normal component of *F*_*R*_ (*= F*_*R*_ cos(*ψ*_*R*_)), of which the vertical component directly contributes to *F*_*comp*_. Because four rollers contribute to the total compensation force, *F*_*comp*_ can now be calculated according to:

$$F_{comp}(\alpha )=4\frac{\cos\left(\psi_{r}\right)\cos\left(\psi_{R}\right)\sin\left(\alpha \right)}{\cos\left(\alpha -\psi_{R}+\psi_{r}\right)}F_{s}$$ (5)

Because of the chosen direction of the friction angles (see Fig 3), Eq (5) corresponds to the *unloading* curve of the mechanism, which is the effective compensation curve.

By combining Eqs (1), (4) and (5) it can be observed that, when *α* = 0, the spring force is at its maximum but the compensation force is zero. When *α* → *π*/2, the spring force is minimal but the compensation force reaches its maximum. In the absolute case of *α* = *π*/2, the mechanism will approach a theoretical asymptote. Because of this asymptote, the mechanism expresses a negative progressive stiffness characteristic, making it suitable for a glove stiffness compensation mechanism.

#### Stabilization

Although rolling link mechanisms show very low values for energy dissipation, they need to be stabilized by a stabilization band [24]. This is done by cross-weaving steel bands through the rolling elements and fixating their ends under tension with micro spot welds (see Fig 2A).

In order to prevent misalignment and asymmetry during operation, the part on which *F*_*res*_ operates (see Fig 2B) is guided between two axes. These axes are fixed on the lower intermediate body and fitted with plain bearings. This same part also prevents the rollers from rolling inwards too much, which can put the mechanism in a form-lock.

#### Materials

All main parts were made of AISI 314 stainless steel. The rollers' radius *r* was taken at 1.9 mm such that a vertical gap of at least 7.5 mm could be obtained–a necessity for the WILMER WHD-4. The contour radius *R* was set to 2.3 mm and width *b* to 7 mm in order to be able to fit the springs.

Two different types of springs were used in order to test the effect of spring stiffness and validity of the model. They were identical in dimensions but varied in material, one stainless steel (T40840) and one spring steel (T30840) from [30]. Both types of springs were able to overcompensate the measured glove stiffness characteristic, having a rated stiffness of 1.35 and 1.57 Nmm^–1^ and pretension of 1.8 and 2.13 N, respectively. The stiffness and pretension were also measured for all springs in order to find their actual mechanical properties.

The stabilization bands were made out of stainless steel. Moreover, they were varied in thickness between 20 and 50 μm to test their effect on the compensation characteristic.

### Data acquisition

Stiffness characteristics were measured of the gloved prosthesis (*F*_*prost*_), the prototype (*F*_*comp*_) and the combination of the two (*F*_*res*_). The latter situation was also tested with different sized cycles at one-third, two-third and 100% of the total possible opening width of the prosthesis, providing insight on the consistency of the mechanism.

A custom-built test bench was used to obtain the stiffness characteristics by measuring absolute force and displacement [9, 10, 13–15]. The direction of force corresponded to the situation as shown in Fig 2B. In the test bench, the measurand was fixed into position and connected to a cable which inflicted displacement. The force at the end of the cable was measured by a load cell (model: B3G-C3-50kg-6B, Zemic, Etten-Leur, The Netherlands) and the position by a linear position transducer (model: LCIT 2000, S/N: J 0069, Schaevitz, Hampton, VA). Both force and position measurements were fed to a data acquisition (DAQ) device (model: NI USB-6008, 12-bit, 10 kS/s, National Instruments, Austin, TX) and into the computer, using LabView version 10.0.1 (National Instruments, Austin, TX) for visualisation and storing of the measured values.

All measurements were repeated five times. Before each series of measurements, the stiffness of connective elements (e.g. cable) was measured separately and eventually corrected for during data processing.

### Data processing

The desired compensation characteristic needed to be based on a generalised shape of the used glove. As a result, each repetition from the glove stiffness measurements was fitted with polynomial and exponential functions. The best fit was chosen based on the adjusted *R*^*2*^ and Root Mean Square Error (RMSE). The fits for every repetition were then averaged to obtain a generalised fit.

The measurements involving the prototype were processed by a moving average. The raw data were divided into separate windows of 0.1 mm covered distance, in which a weighted average was calculated using a Gaussian function. Also, using the same weighting factors, a weighted standard deviation was calculated.

For all tests, the amount of input energy was determined by calculating the area beneath the loading curve. Hysteresis was determined by calculating the area enclosed by the cycle. The efficiency of the RSCM combined with the prosthesis was determined by calculating the ratio between the loading and unloading curve.

All data processing was performed in Matlab 2010b (Mathworks, Natick, MA).

## Results

Raw data that is used to generate the results is supplied as supporting information in S1 Dataset.

### Gloved prosthesis

**Fig 4:**
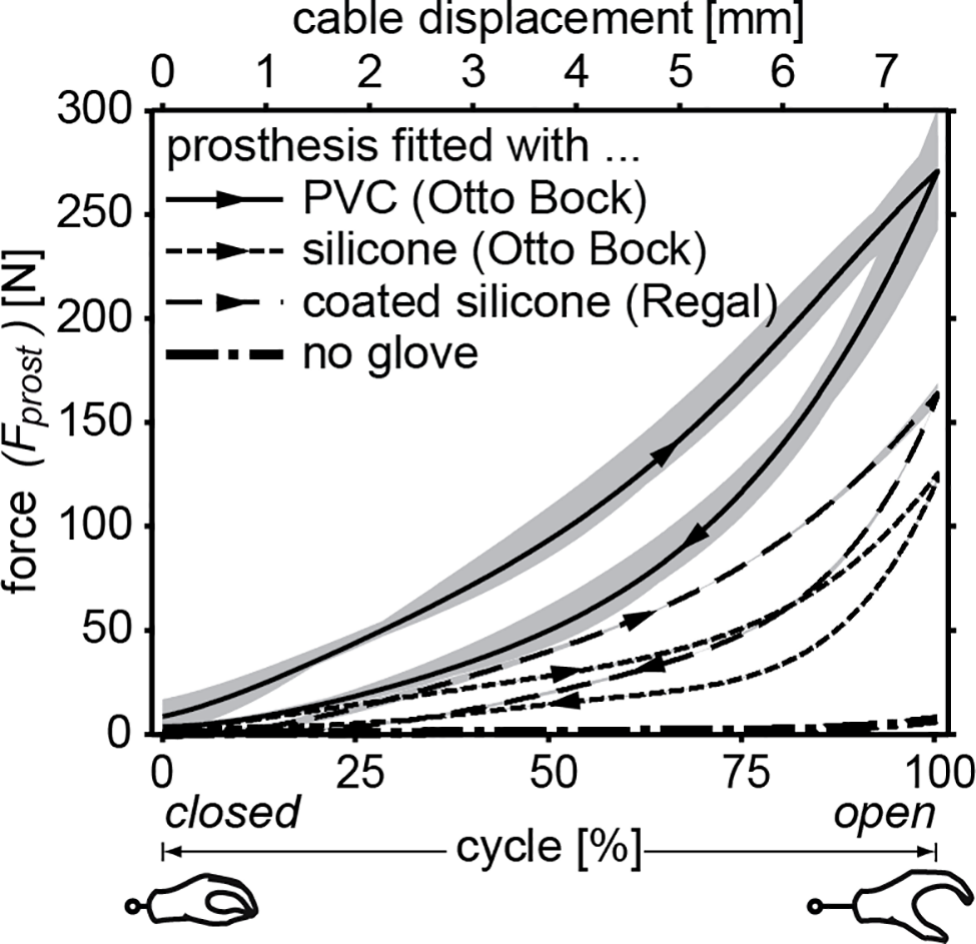
Stiffness characteristics of a prosthesis fitted with different gloves. Showing the average fit (black) compared to the range of different fits (grey) for all types of glove material, as well as for the ungloved prosthesis. The area beneath the loading curve is equal to required input energy (*E*_*in*_), area enclosed by both curves is equal to the hysteresis (*E*_*hyst*_). Average energy values are for PVC (Otto Bock): *E*_*in*_ = 833 Nmm, *E*_*hyst*_ = 259 Nmm, for silicone (Otto Bock): *E*_*in*_ = 277 Nmm, *E*_*hyst*_ = 105 Nmm, for coated silicone (Regal): *E*_*in*_ = 388 Nmm, *E*_*hyst*_ = 133 Nmm.

For each separate glove material fitted on the prosthesis, a fifth-order polynomial proved to provide for the best fit of the measured stiffness characteristics (adjusted *R*^*2*^ > 0.99, RMSE < 5 N). The fitted characteristics for all types of gloves that were used are shown in Fig 4, where distinctive loading and unloading curves can be seen. The required input energy (*E*_*in*_) to open the gloved device ranged between 277–833 Nmm. The hysteresis (*E*_*hyst*_) ranged between 105–259 Nmm. The ungloved WILMER WHD-4 never showed forces higher than 10 N, confirming that the addition of the cosmetic glove has a dramatic effect on the necessary operation forces.

The shapes and outcome measures are comparable to those of previous studies [12–14]. Here, the pure silicone gloves from Otto Bock show the lowest energy values and require a peak activation force of approximately 120 N. Resultantly, these were used as a reference point to define the desired stiffness characteristic.

### Prototype

**Fig 5:**
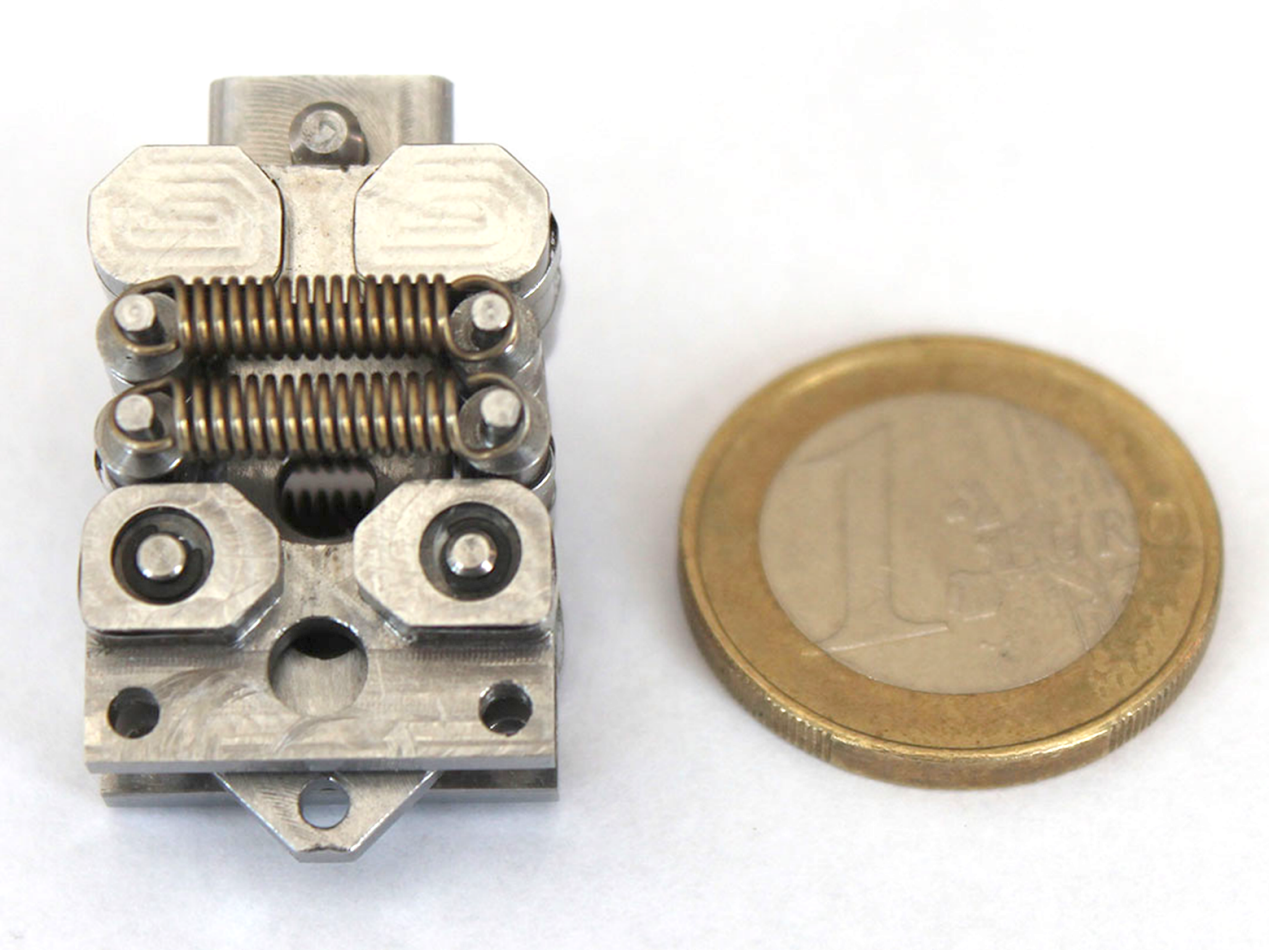
The manufactured RSCM prototype. Displayed alongside a Euro coin [Ø 23.25mm] for scale.

The resulting prototype is shown in Fig 5, where protrusions were added to the overall shape to guide the stabilization bands. The outer dimensions (length×width×depth) are equal to 33×18.6×19 mm when the springs are relaxed (see Fig 5) and 33×26.2×19 mm when the springs are fully tensed. The total mass of the mechanism is 26 g.

The springs used appeared to have different mechanical properties than reported by manufacturer. Stainless steel springs showed an average stiffness of 1.05 Nmm^–1^ and pretension of 0.63 N (versus rated 1.35 Nmm^–1^ and 1.8 N), spring steel springs showed a stiffness of 1.18 Nmm^–1^ and pretension of 0.86 N (versus rated 1.57 Nmm^–1^ and 2.13 N).

**Fig 6:**
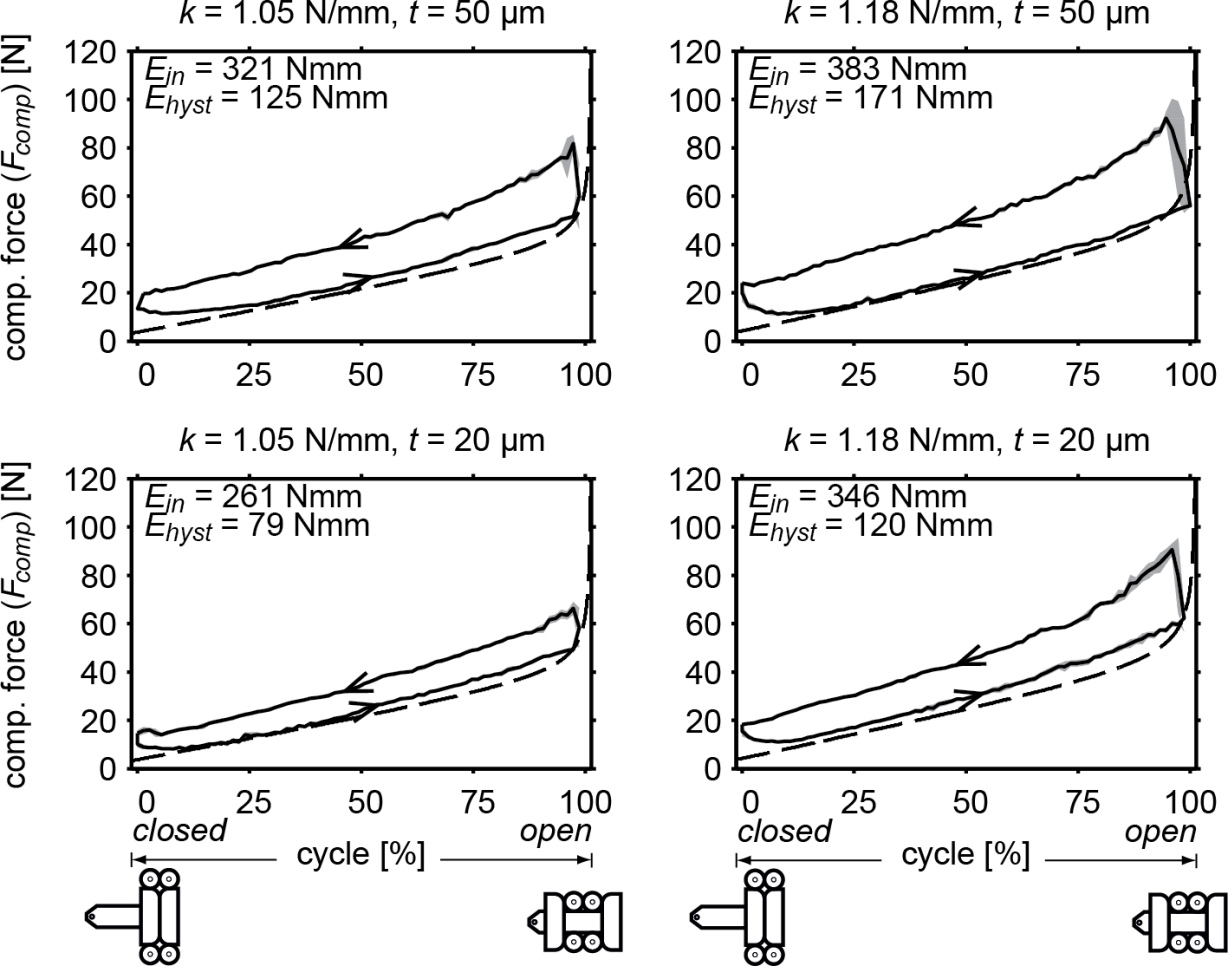
Absolute compensation force of the prototype. Showing the averaged measured force (solid) with standard deviation (grey) and as predicted by the model (dashed). Input energy and hysteresis are also shown for measured values. Both spring stiffness (*k*) and stabilization band thickness (*t*) were varied. Arrows indicate the direction of the curves, distinguishing loading and unloading curves.

The results for the RSCM's absolute compensation characteristic compared with the model output are presented in Fig 6. All graphs show that the model prediction of the unloading curve is indeed largely comparable to the unloading curve of the measured values, where an increase in spring rate results in increased overall stiffness and higher reaction forces. Increasing stabilization band thickness has a similar effect, but was not included in the model and therefore only affects the measured values. The measured values show a small offset and a large hysteresis loop due to the much higher loading curve. As a result, the magnitudes of input energy (*E*_*in*_) and hysteresis loop (*E*_*hyst*_) are large and range between 261–383 Nmm and 79–171 Nmm, respectively. Both energy parameters increase with spring rate and stabilization band thickness.

**Fig 7:**
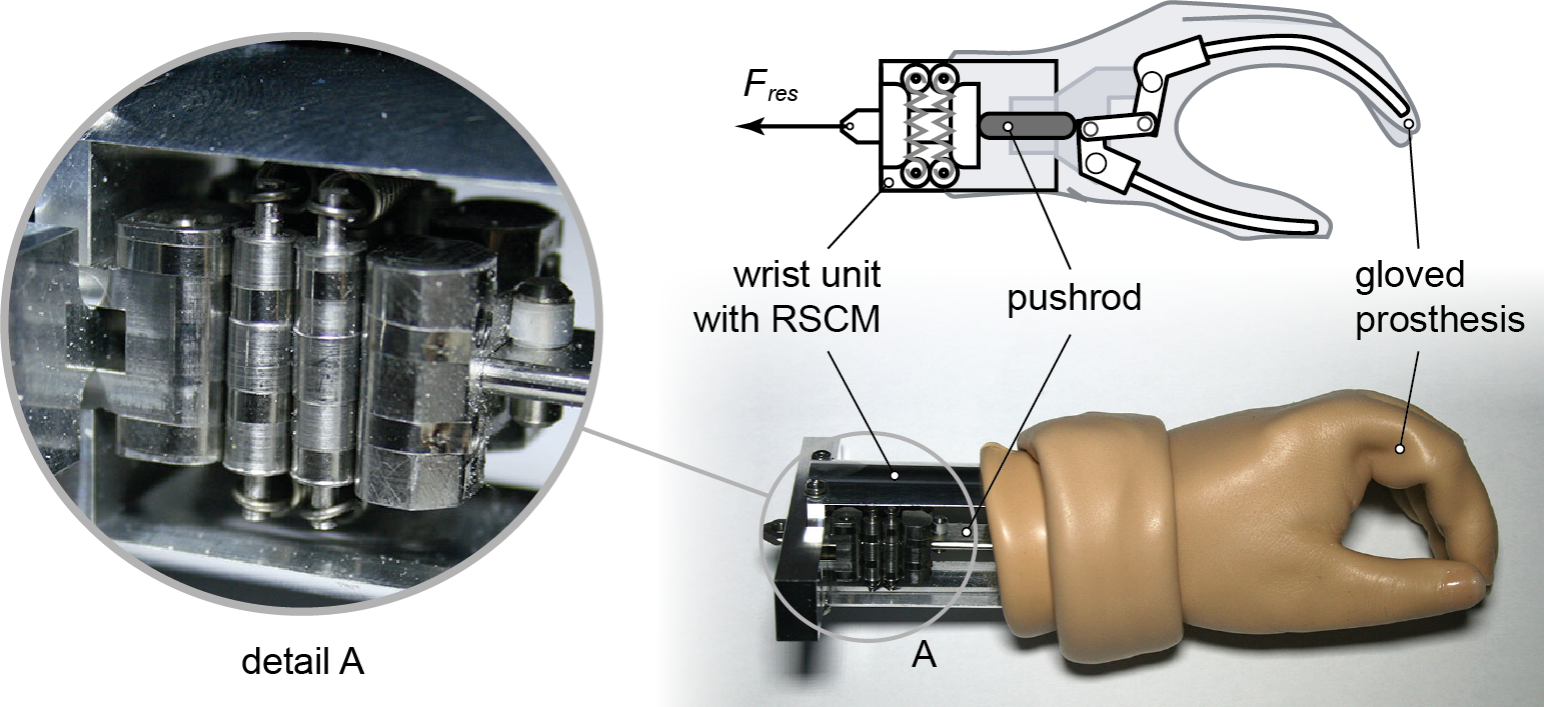
The compensated hand prosthesis. A photo and corresponding illustration of the RSCM in combination with a gloved hand prosthesis. The RSCM was fixed into a wrist unit and pushed against the gloved prosthesis using a pushrod.

**Fig 8:**
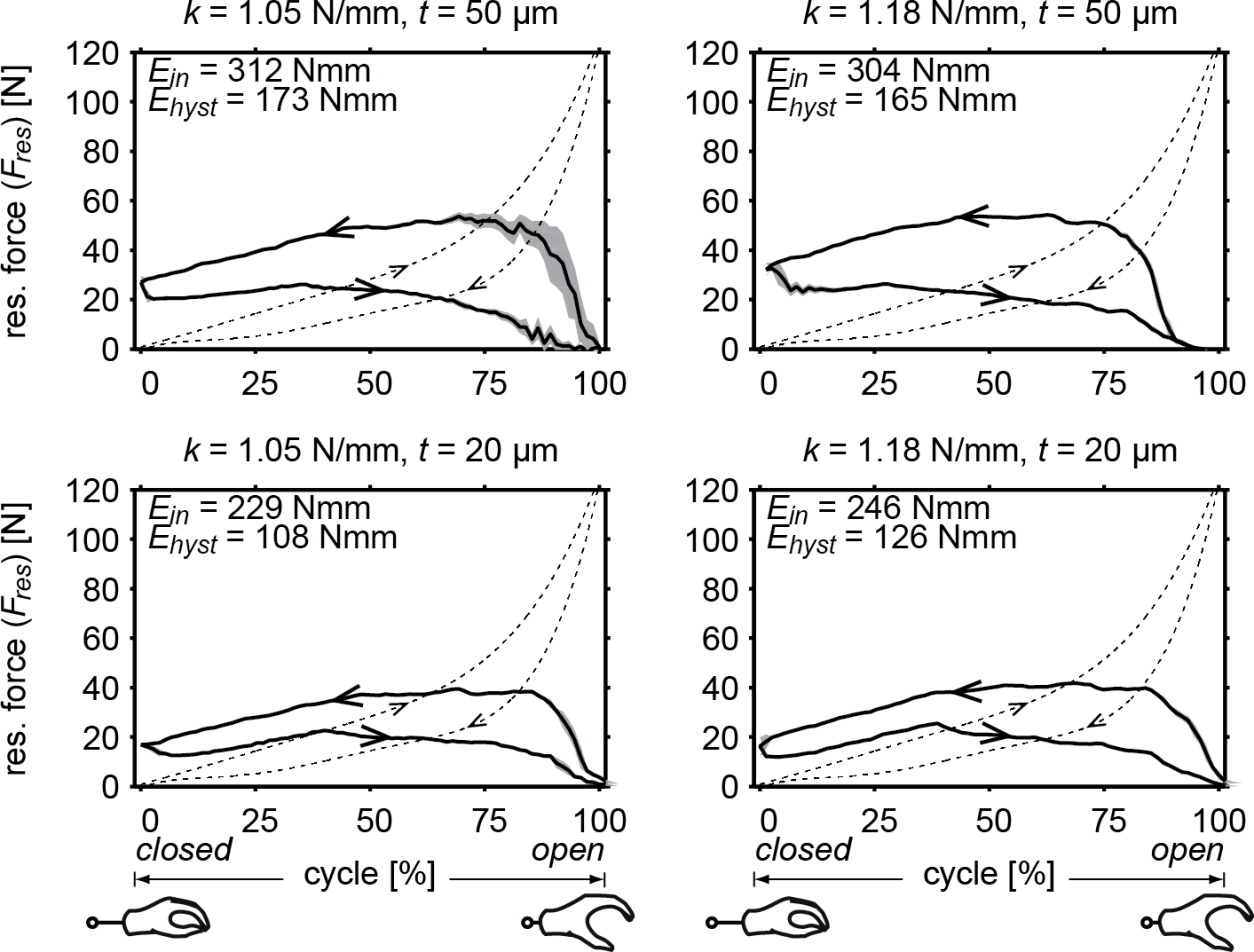
Resultant force of the compensated gloved prosthesis. Showing the averaged measured force (solid) with standard deviation (grey) and compared to the original gloved prosthesis (dotted). Input energy and hysteresis are also shown for the compensated prosthesis. Both spring stiffness (*k*) and stabilization band thickness (*t*) were varied. Arrows indicate the direction of the curves, distinguishing loading and unloading curves. Notice how the direction of the curves have been reversed, creating a voluntary closing prosthesis.

The RSCM combined with the gloved prosthesis is shown in Fig 7. The absolute resultant forces of this combined system are shown in Fig 8, where a comparison is provided with the original—uncompensated—gloved prosthesis. It can be seen that the operation forces are not necessarily lowered, but the peak forces are reduced. In the case where *k* = 1.05 Nmm^–1^ and *t* = 20 μm, the peak force is reduced the most to below 40 N. At *k* = 1.18 Nmm^–1^ and *t* = 50 μm, the mechanism showed a small amount of under-compensation at around full opening width and could not go through the complete cycle–giving lower energy values. The resultant values for input energy (*E*_*in*_) range between 229–312 Nmm and for hysteresis (*E*_*hyst*_) between 108–173 Nmm. These values include the hysteresis of both the gloved prosthesis and compensation mechanism and are therefore higher in magnitude. They are, however, not equal to the addition of the two, indicating a dependence on operation force.

Thinner stabilization bands bring the characteristic closer to the model—both in shape and in absolute values—by reducing input energy and hysteresis. Stabilization bands of 50 μm give the RSCM an average efficiency of 58%. Combined with the gloved prosthesis, it is able to reduce maximum resultant forces down to 55 N with a combined efficiency of 45%. Bands of 20 μm, on the other hand, are able to reduce them down to 40 N, where the RSCM shows an average efficiency of 68% and a combined efficiency with the gloved prosthesis of 52%.

**Fig 9:**
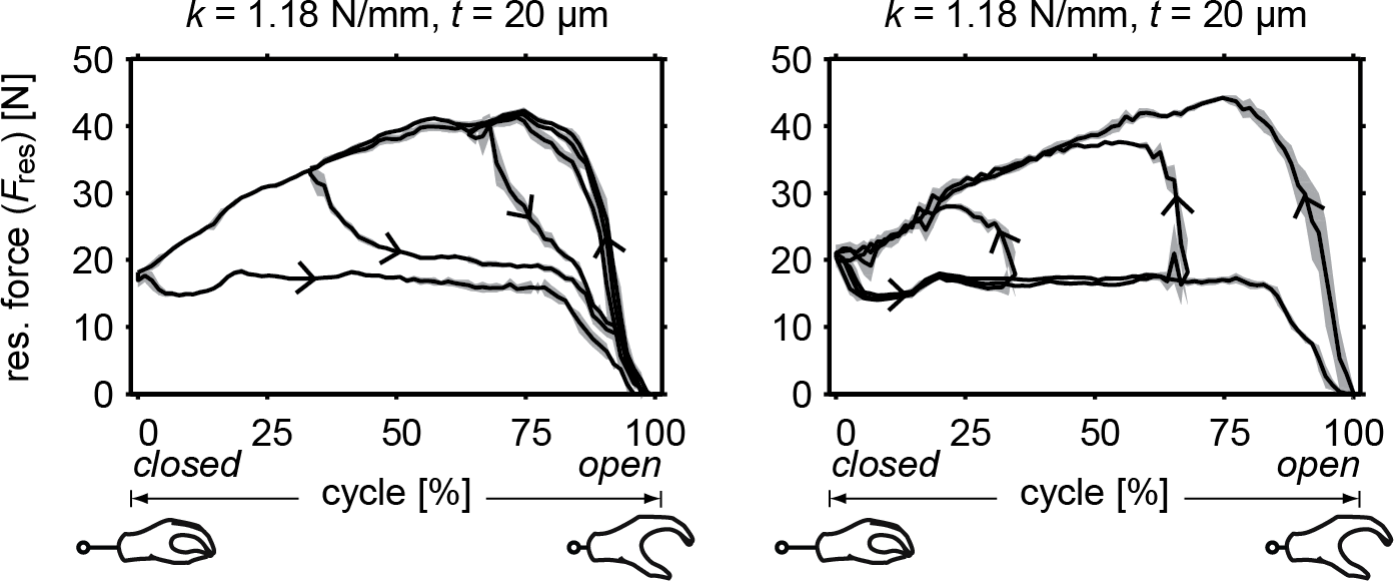
Resultant force of the compensated gloved prosthesis at different cycles. Showing the averaged measured force with standard deviation of the gloved prosthesis with compensation mechanism, undergoing different sized cycles at one-third, two-third and 100% of the total opening width. The left figure starts with an open hand (resting position), closing the hand at the different widths. The right figure starts with a closed hand, opening the hand at the different widths.

Fig 9 shows the compensated gloved prosthesis undergoing different sized cycles at one-third, two-third and 100% of the total opening width of the prosthesis. As the prosthesis is closed at different widths (left figure), the loading curves remain the same but the unloading curves take different paths. However, as the prosthesis is opened at different widths (right figure), both loading and unloading curves remain to take the same path.

## Discussion and conclusion

The RSCM is successful in having a progressive negative stiffness characteristic and is therefore suitable as a novel compensation mechanism. However, the mechanism's performance is largely restrained by the stabilization bands: their thickness causes large hysteresis loops and inaccuracies during assembly may cause misalignment of parts. Nonetheless, it is able to reduce operation forces of a gloved prosthesis and even reverse its working principle with overcompensation, making it possible to turn a voluntary opening device into a voluntary closing device.

A toddler-sized prosthesis fitted with an Otto Bock silicone glove shows a peak force of 120 N and input energy of 277 Nmm. It is shown that the RSCM is able to reduce the peak force down to the comfortable limit of 40 N [11] and lower the input energy down to 229 Nmm. This is done by using springs with a stiffness of 1.05 Nmm^–1^ and stainless steel stabilization bands with a thickness of 20 μm. Compared to the large reduction in peak force, the input energy is only slightly reduced, indicating that the current prototype mainly redistributes the necessary input energy.

The loading and unloading curves of the resultant characteristic are predictable and mostly independent on their previous paths. However, the unloading curve towards a fully opened hand can slightly differ. This may be blamed on the viscoelastic properties of the cosmetic glove, i.e. not returning to a fully closed (resting) state.

The small dimensions of the RSCM allow it to be used inside the wrist of a toddler-sized prosthesis. It requires a minimum inner diameter of 33 mm, which may easily fit into a 38 mm diameter wrist. The RSCM can also be integrated with the prosthesis mechanism as part of a pushrod, further reducing the length of the mechanism. The low mass minimally affects the overall mass of the prosthesis, bringing it to a total of 95 g.

The performance of the mechanism is mostly influenced by the stabilization bands and misalignment of parts. Specifically, thicker stabilization bands add more rigidity to the system, introducing a higher stiffness due to elastic deformation and energy dissipation due to irreversible deformation. This is reflected by the increasing values for input energy (*E*_*in*_) and hysteresis (*E*_*hyst*_) as band thickness (*t*) increases. Thinner bands, however, are increasingly difficult to assemble. Their fixations (spot welds) become weaker, inaccuracies are more likely to occur and less pretension can be added. This causes the mechanism to become more sensitive to external factors and individual parts to become misaligned, resulting in non-parallel axes of the rolling elements–one of the larger sources of rolling friction [24]. Improved and more automated assembly strategies should reduce these side-effects, which would allow for thinner bands without compromising the robustness of the mechanism.

Misalignment of parts is not only caused by quality of assembly of stabilization bands. It was also observed that the direction of pulling force from the test bench was not always perfectly in line with the mechanism, which caused the rolling elements to become misaligned. Its magnitude is dependent on the operation force, hence it was much lower when the RSCM was tested in combination with the prosthesis. This is confirmed by the fact that the resultant hysteresis is not equal to the hysteresis of the gloved prosthesis added with the RSCM's hysteresis. By adding an intrinsic alignment of the parts, for example by rolling in grooves, this effect can be reduced.

An additional practical improvement to the design would be to increase the rollers radius *r*, which further prevents a form-lock of the mechanism when *α* → *π*/2. Moreover, this increases the working space of the RSCM and even becomes larger than required. This allows the relative position of the RSCM to be tweaked on-the-spot, increasing its range of applicability and tolerance towards inaccuracies.

Apart from the presented configuration, i.e. the used geometry and springs, other configurations are also possible. In general, the ratio between the roller's radius *r* and contour radius *R* defines the shape of the RSCM's negative stiffness characteristic. Even non-circular shapes are possible to implement. The springs and stabilization bands then determine the magnitude and attended losses. This modular principle makes the RSCM suitable to be designed for other types of gloves and even other applications.

In conclusion, the RSCM is a novel negative stiffness element with a large possible area of application. At current stage, its efficiency leaves something to be desired. However, it is believed that its performance can be further increased by improving stabilization band assembly and constraining out-of-line movements.

## Supporting information

S1 Dataset Raw measurement data. (ZIP)
